# Enhancing Therapist Training in the Delivery of Exposure Therapy for Individuals with Anxiety Disorders Using Virtual Reality Simulation: Randomized Feasibility Trial

**DOI:** 10.2196/80087

**Published:** 2026-05-21

**Authors:** Joshua Kemp, Ariella Rosen, Hyungjin Kim, Margo Adams Larsen, Kristen Benito, Jennifer Freeman, Jason Machan, Peter Tuerk

**Affiliations:** 1 Department of Psychiatry and Human Behavior, Alpert Medical School, Brown University, Providence, RI, United States; 2 Pediatric Anxiety Research Center, Bradley Hospital, Brown University Health, 1011 Veterans Memorial Pkwy, Riverside, RI, 02915, United States, 1 4014321466; 3 Virtually Better Inc, Decatur, GA, United States; 4 Department of Biostatistics, Brown University Health, Providence, RI, United States

**Keywords:** virtual reality, exposure therapy, anxiety disorders, training, simulation

## Abstract

**Background:**

Exposure-based cognitive behavioral therapy is among the least used evidence-based practices for anxiety disorders in routine care. Providers’ negative beliefs about exposure (eg, fears of harm or intolerability) are a major barrier. Experiential methods can reduce these beliefs but are limited by accessibility, standardization, and fidelity. Virtual reality (VR) offers a scalable way to deliver standardized experiential practice. Guided by an “exposure to exposure” (E2E) framework, we conceptualized VR training as an exposure intervention targeting therapists’ own anxious beliefs about exposure.

**Objective:**

This feasibility study examined a VR-based exposure training program (SET-VR) (1) to evaluate usability and effects on therapist learning targets (knowledge, self-efficacy, attitudes) and (2) to test whether a high-immersion head-mounted display (HMD) format provides added benefit over a lower-immersion desktop format. Eligibility included holding an active caseload.

**Methods:**

Eligible clinicians (ie, aged >21 years with an active caseload; n=41) completed a 4-hour didactic workshop on exposure and were randomized (1:1, blinded) to the desktop or HMD condition. In the experiential phase, therapists delivered 3 rounds of exposure with a virtual patient. They titrated exposure intensity (increase, decrease, continue as is) at fixed decision points based on state-dependent visual (character animations) and auditory (prerecorded verbalizations) cues reflecting the patient’s distress. Exposure knowledge, self-efficacy, and beliefs about exposure were measured at baseline, post-didactic, post-experiential, and follow-up. Participants also rated the acceptability, usability, and authenticity of the program.

**Results:**

Both groups (desktop and HMD) showed significant improvement in exposure knowledge (*d*=0.52, *P*=.006; *d*=0.58, *P*=.002), self-efficacy (*d*=0.88, *P*<.001; *d*=1.36, *P*<.001), and beliefs (*d*=0.61, *P*=.001; *d*=1.05, *P*<.001) from baseline to post-didactic training using binomial generalized estimating equations. There were no significant differences between the low- and high-immersion groups on any measure after didactics. Both groups demonstrated significant improvement in exposure self-efficacy (*d*=0.66, *P*<.001; *d*=0.93, *P*<.001) and beliefs (*d*=0.46, *P*<.01; *d*=0.66, *P*<.001) from post-didactic to post-experiential. Both groups gave positive ratings for acceptability, usability, and authenticity. No adverse events or side effects were reported.

**Conclusions:**

In this feasibility randomized controlled trial, an E2E-guided VR training program produced promising improvements in therapists’ self-efficacy and negative beliefs about exposure beyond gains from didactic training alone. This work is innovative in testing immersion as a dose parameter while also applying an explicit framework (E2E) to target a key mechanism (ie, therapist beliefs) in the underuse of exposure therapy. Compared to prior VR training studies focused on skills and knowledge acquisition, our findings support the standardization of an emotionally engaging exposure practice context that shifts therapist-level mechanisms linked to actual delivery. The lack of clear advantages for HMD over desktop VR suggests that lower-immersion, more scalable implementations may provide a sufficient experiential “dose.” Larger, more diverse trials are needed to confirm effectiveness and determine the real-world impact of VR-based exposure training on access to evidence-based care.

## Introduction

Anxiety disorders are among the most common mental health conditions in children and youth, impacting 1 in 5 children since the COVID-19 pandemic [1]. Symptoms in youth can manifest as uncontrollable worry, fear, and hyperarousal, leading to a cascade of developmental consequences and pervasive disability in adulthood [2,3]. Cognitive behavioral therapy (CBT) is a highly effective treatment for addressing anxiety in children and is regarded as the first-line treatment [4]. Within CBT for anxiety, exposure therapy has gained extensive empirical support as a frontline intervention across anxiety disorders, including obsessive-compulsive disorder and posttraumatic stress disorder [5].

Despite robust empirical support, exposure-based CBT remains one of the least utilized evidence-based practices in typical practice settings, with as few as 7% of patients reporting receiving exposure therapy for their anxiety disorder [6]. A leading barrier to its dissemination is provider access to training, with only 12% to 28% of therapists reporting having received training to provide exposure therapy [7]. Another barrier is therapists’ willingness to use exposure therapy after receiving training. Surveys suggest that a majority of therapists who have received training seldom use exposures in practice [7,8]. The current “gold standard” for exposure training involves a workshop, an accompanying manual, and clinical supervision [9,10]. Unfortunately, research suggests that this training approach may not be sufficient to prepare therapists to deliver exposure therapy. One study randomizing therapists to different “doses” of training showed that only 54% of clinicians receiving the highest “dose” of training reached proficiency levels [10]. Most training approaches are purely didactic in nature, with the goal of transferring knowledge about exposure and its delivery process; however, research consistently demonstrates that exposure knowledge is not predictive of uptake and quality delivery [9,11,12]. This suggests that current training approaches lack the effectiveness to promote the necessary levels of proficiency and confidence for providers to put exposure therapy into practice. Training innovations focused on the accessibility and methodology of exposure therapy training are necessary to overcome this glaring research-to-practice gap.

A key area unaddressed in conventional training that may explain the underutilization of exposure following training is therapists’ negative beliefs about exposure. Researchers have found that therapists’ decision to not use exposure therapy is linked to beliefs about its dangerousness or risk of negative events [13]. Such beliefs include concerns about symptom exacerbation [14], patient decompensation [15], direct patient harm [16], and eventual treatment dropout. However, serious negative consequences from exposure therapy are incredibly rare and comparable to those of other therapeutic modalities [17]. These negative beliefs are also associated with suboptimal delivery when exposure is used (ie, deviation from the prolonged and intense delivery recommended by exposure theorists and treatment manuals) [18]. This includes the introduction of breathing strategies [19], allowing the use of safety behaviors and premature termination of exposures [20], all of which negatively impact the efficacy of exposure therapy [21]. Given the well-documented role of negative beliefs in stifling both the use and optimal delivery of exposure therapy, ideal training in exposure therapy must include strategies to target this barrier.

Training studies of exposure therapy have shown that experiential learning (ie, partner- and self-exposure) can be an effective way to reduce therapists’ negative beliefs [22]. This is consistent with the benefits of experiential learning observed in other domains of medical training [23]. Given the similarities between therapists’ negative beliefs about using exposure and clients’ anxious beliefs about engaging with feared items, and the well-established efficacy of exposure as a process for reducing anxious beliefs, it is logical to approach experiential tasks during exposure training as E2E to elicit and ameliorate therapists’ negative beliefs [24]. Based on this logic, studies have demonstrated the benefits of incorporating experiential activities, such as role-playing, to enhance training [25]. While this is a step toward improving the quality of exposure therapy training, it faces challenges in accessibility, standardization, and fidelity to real-life experiences that limit optimization. Building on this E2E framework [24], we conceptualized the training exercises themselves as a form of exposure intervention directed at therapists’ own anxious beliefs about exposure. In this view, a key design question is how closely the training context must approximate real exposure sessions with distressed patients for therapists to experience corrective learning about the safety and tolerability of exposure. VR-based simulations provide a way to systematically manipulate this “experiential dose” of E2E by varying the level of immersion while holding didactic content constant.

Virtual environments (VEs) and virtual reality (VR) offer a promising solution to further enhance exposure training via experiential learning. VEs are computer-created environments with objects that represent real-life counterparts and allow users to interact with these simulations in real time as though they were real [26]. VR involves the display of computer-generated environments in a 3D manner, which allows the user to experience immersive, multisensory interactions in real time [26]. VR has unique advantages in its representativeness of real clinical situations, individualization, replicability, and economic scalability—all of which currently limit the efficacy and reach of conventional role-playing and other forms of experiential learning [27]. Leveraging these strengths, the medical education field has recently made efforts to integrate VR into training with positive outcomes [23]. Emerging evidence suggests that VR may also be an effective tool for improving skills and attitudes pertinent to the treatment of mental health concerns. A study of mental health nursing trainees demonstrated VR’s effectiveness in improving knowledge, skills, and attitudes [28]. A recent systematic review of VR in psychiatric education found several studies showing improved knowledge and clinical skills for participants across a range of conditions [29]. VEs and VR show great promise for solving exposure training’s dual challenges of accessibility and provider-level barriers via E2E for clinicians.

There has been growing research into how different aspects of a learner’s experience within the virtual environment impact their learning outcomes. Presence, the feeling of “being there” in the virtual setting [30], has been identified as a moderator of increased engagement [31], emotional activation [30], and social attitude change [32]. Embodiment, the sense of occupying the virtual avatar controlled by the user [33], has been shown to generally improve engagement and outcomes especially for retention [34]. Generally, interacting with virtual environments via hardware, such as head-mounted display (HMD), has shown higher levels of presence and embodiment than use through computer monitors or hand-held screens [35]. Because the research on VR-based delivery of mental health training is in its nascency, studies have focused on whether incorporating VR enhances learning outcomes compared to conventional nondigital methods [27,36], and this study team found no studies that directly compare across different immersive experience conditions. This limits our ability to understand how VR-specific variables may impact learning outcomes. Thus, further investigation into how these variables impact psychotherapy training will be a key step in optimizing the effectiveness and implementation of virtually delivered mental health training. The goal of this study was to evaluate the feasibility of a theory-driven, simulation-based E2E training program and to obtain initial evidence that supplementing didactic exposure training with experiential VR can shift key therapist-level learning targets. Specifically, we tested the recently developed Simulated Exposure Trainer (SET-VR) in a randomized feasibility trial that compared 2 delivery modalities differing in levels of immersion (low: desktop computer; high: HMD), conceptualized as 2 experiential doses of E2E practice. Our primary questions were (1) whether SET-VR would be usable and acceptable to clinicians and produce additional improvements in exposure knowledge, perceived self-efficacy, and therapist beliefs about exposure beyond a standard didactic workshop, and (2) whether high-immersion HMD VR would produce any incremental benefit over lower-immersion desktop delivery on these mechanism-based outcomes and perceived realism. It was hypothesized that both modalities would meet an a priori benchmark for usability.

## Methods

### Participants

This National Institute of Mental Health–funded (R41MH131229) study aimed to recruit a final sample of 40 clinicians. Of the 42 participants enrolled in the therapist training trial, one withdrew after consenting due to scheduling difficulties. The remaining clinicians (N=41) completed all stages of the training trial, including follow-up. Clinicians were recruited from a variety of settings between November 2023 and April 2024. Recruitment methods included convenience sampling (recruiting from the pool of clinicians at Bradley Hospital), offering training to clinicians on a waitlist to participate in nonexperimental CBT training at Bradley led by the first author, and targeted email advertising to social workers, private-practice clinicians, and providers affiliated with a local mental health practice. The eligibility criteria for the study included a bachelor’s level of education (or higher), the ability to come to Bradley Hospital for in-person experiential training, and the ability to deliver clinical care to patients in a professional capacity.

The majority of the therapist participants (37/41, 90%) were White and non-Hispanic. Four out of 41 (10%) therapists reported Hispanic or non-White identities. A majority (35/41, 85%) also identified as female. Participants had a variety of degree types, including doctorate (n=5), master’s (n=27), and bachelor’s-level (n=9) degrees. Licensure types include clinical psychologists (n=4), mental health counselors (n=13), social workers (n=13), and either graduate students or other (non-licensed) clinical roles (n=11).

### Measures

#### System Usability Scale

The System Usability Scale (SUS) is a 10-item questionnaire that asks participants to rate a website or program across areas, including ease of use, intuitiveness, functionality, and user confidence on a scale of 1 (“strongly disagree”) to 5 (“strongly agree”) [37]. Raw total scores are converted to a 0‐100 scale, with values >70 indicating average or better usability and >80 indicating excellent usability.

#### Virtual Patient Evaluation

The Virtual Patient Evaluation (VP Eval) consisted of 12 items rated on a 5-point Likert scale (“strongly disagree” to “strongly agree”), as well as 3 open-text responses [38]. The first 6 items focused on the authenticity of the therapist’s engagement with the virtual patient (eg, “While working with the virtual patient, I felt I had to make the same decisions a therapist would make in real life”), while items 7 to 12 assessed SET-VR as a training tool and the extent to which it helped the therapists feel ready to engage with an actual patient.

#### Therapist Negative Beliefs about Exposure Scale

The Therapist Negative Beliefs about Exposure Scale (TBES) assesses the extent to which therapists agree with 21 negative beliefs about exposure therapy (eg, “most clients have difficulty tolerating the distress exposure therapy evokes”). Items are rated on a 5-point scale from 0 (“disagree strongly”) to 4 (“agree strongly”) [19]. Possible scores range from 0 to 84, with a higher score indicating more negative beliefs about exposure. The TBES is the primary outcome measure for assessing target engagement.

#### Exposure Knowledge

We condensed the original 49-item measure into 12 multiple-choice items that best fit the didactic content of the training (eg, “Why is it important to block avoidance during exposure tasks?”) [20]. Items assessing procedures or content not included in this training were removed, leaving a subset aligned with the material taught in this trial. The same subset of items was used in the preceding study to this trial [24]. The total score is the percentage of correct answers out of 100.

#### Exposure Self-Efficacy

This is a 27-item measure of therapists’ confidence in delivering exposure therapy [20]. The first 8 items focus on the therapist’s ability to help patients learn skills related to exposure (eg, “I feel confident in my ability to help my clients identify how avoidance is maintaining their fear”), and the remaining 19 items assess the therapist’s confidence with implementing various aspects of exposure (eg, “I feel confident in my ability to conduct imaginal exposure”). Items are rated on a scale ranging from 1 (“not confident”) to 5 (“very confident”). This measure has demonstrated high internal consistency and predictive validity in determining the frequency of self-reported clinical use of exposure therapy.

#### Training Acceptability Rating Scale

The Training Acceptability Rating Scale (TARS) is a measure of therapists’ satisfaction [39]. The first 6 items, rated on a scale of 1 (“strongly disagree”) to 6 (“strongly agree”), assess the acceptability of the training program, and the following 9 items ask therapists to rate the training’s perceived utility on a scale of 1 (“not at all”) to 4 (“a great deal”). Item means for acceptability and effectiveness subscales are calculated separately. This measure has good psychometric properties.

### Procedures

#### Overview

The total training duration was 6 hours, comprising a 4-hour didactic learning course delivered over Zoom and a 2-hour in-person experiential training that took place on-site at Bradley Hospital. All clinicians (n=41) received the same didactic training. After the online training workshop, participants were scheduled for the second portion of training and randomly assigned to either the VR (high immersion; n=20) or computer (low immersion; n=21) experiential learning conditions. Participants were allocated to the conditions in a 1:1 ratio using random number generation and the randomization tool in REDCap (Research Electronic Data Capture). The randomization of each participant took place as closely as possible to the start of experiential training, which prevented the research team from having advance knowledge of the assignments. All 3 learning measures (ie, exposure knowledge, self-efficacy, and negative beliefs) were administered at baseline (T0), post-didactic (T1), post-experiential (T2), and 1-month follow-up (T3) to allow a direct comparison of changes in learning over time (Table 1).

**Table 1.** Key outcome measures and timepoints for delivery in the SET-VR^a^ therapist training trial.

Measure	Baseline (T0)	Post-didactic workshop ( T1)	Post-experiential learning with SET-VR (T2)	Follow-up (T3)
Demographics	✓			
TBES^b^	✓	✓	✓	✓
Exposure knowledge	✓	✓	✓	✓
Self-efficacy	✓	✓	✓	✓
System usability scale			✓	
VP^c^ evaluation			✓	
TARS^d^			✓	

a SET-VR: Simulated Exposure Trainer-Virtual Reality.

b TBES: Therapist Negative Beliefs about Exposure Scale.

c VP: virtual patient.

d TARS :Training Acceptability Rating Scale.

#### Didactic Workshop

The online workshop took place before randomization and consisted of several modules outlining the main components of exposure therapy and its delivery. The training modules included content areas such as diagnostic review, psychoeducation, functional assessment, exposure design and selection, exposure delivery, and exposure therapy troubleshooting. All explicit exposure-related knowledge (eg, key concepts, principles, and procedures) was delivered during this workshop and was identical for both conditions; the subsequent experiential component did not introduce any new didactic content. To accommodate therapists’ schedules and the staggered nature of recruitment, a total of 8 didactic trainings were held, consisting of approximately 5 individuals per round. All didactic trainings were led by Dr Kemp, and each cohort covered the same course material.

#### Experiential Component (SET-VR Training Scenario)

##### Overview

Experiential training occurred 1 to 2 weeks after completing the didactic portion. Participants were blinded to their randomized condition until the start of their experiential training, at which point trainers disclosed whether they would be using a computer or a VR headset for the task. Given the nature of the task, further blinding was not possible. Participants in both conditions experienced the same SET-VR simulated training scenario (Table 1); only the delivery modality (VR vs computer) differed. Experiential training was conducted one-on-one with a member of the research team (JK or AR). In all, the SET-VR training experience consisted of an introductory sequence orienting users to the program structure and navigation, followed by simulated exposure therapy delivery that consisted of 9 separate decision points and then a feedback module that provided targeted feedback about therapists’ decisions during delivery. The 9 decision points were implemented using a simple branching decision tree, such that the exposure options available at each point depended on the therapist’s immediately preceding choice. Participants repeated the full training scenario 3 times within the 2-hour sitting but were allowed to fast forward through the introductory sequence after their first time using the program. The VR component was designed as a purely experiential practice phase in which therapists applied the exposure procedures learned in the workshop with a simulated patient; no additional exposure-related knowledge content was provided during this phase. A video overview of the SET-VR program can be obtained by contacting the corresponding author.

##### Introductory Sequence

The program began with a 9-slide introductory sequence that welcomed the user to SET-VR, provided relevant background information to orient the therapist to the virtual patient’s presenting concerns, introduced the Subjective Units of Distress Scale (SUDS), and demonstrated how to use “in-game” controls to ask for SUDS ratings and modify exposure difficulty. The sequence concluded with therapists selecting an initial exposure task for the virtual patient from a preconstructed hierarchy of 10 exposure tasks, which was ordered from the most difficult task at the top (eg, “touch shoe to face”) to the least difficult at the bottom (eg, “look at shoe”). The initial exposure task could be selected from anywhere on the hierarchy.

##### Simulated Treatment Delivery

**Figure 1.**
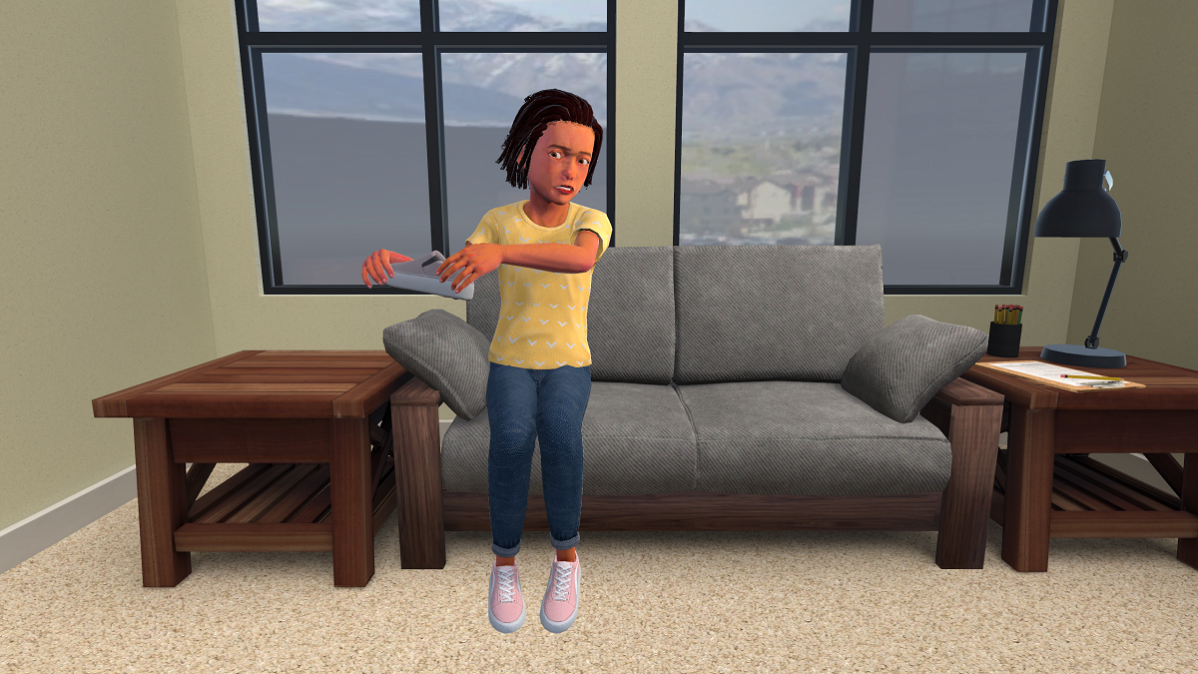
Screen capture of the virtual patient guided by a therapist learner during SET-VR training.

After initiating the first exposure task, the therapist progressed through a fixed sequence of 9 decision points (Figure 1). Beginning with the initial task selection on the hierarchy, the simulation implemented simple branching logic. At each subsequent decision point (occurring every 45 seconds), therapists chose whether to increase exposure difficulty (select an item higher on the hierarchy), decrease the difficulty (select a lower item), or keep it the same (continue with the same exposure task). When raising or lowering the difficulty level of an exposure, available options were restricted to tasks within 2 hierarchy positions above or below the current task. This constraint prevented unrealistic, large jumps in exposure intensity and subtly guided learners toward the gradual titration that characterizes optimal exposure delivery. Throughout the experience, prerecorded verbalizations from the virtual patient played every 15 seconds, and both verbalizations and patient animations were keyed to the patient’s current distress state. The underlying subjective units of distress (SUDS; 0‐10) value was automatically updated by the program, decreasing by 1 point approximately every 2 minutes of continuous exposure to approximate the natural process of habituation during effective exposure sessions rather than being manually adjusted by an instructor. Therapists could sample the virtual patient’s distress level throughout the experience by clicking the SUDS button in the bottom right corner of the screen.

##### System Implementation

The SET-VR app was developed in Unity (C#) and deployed in both immersive VR and desktop configurations using the same core application logic. In the high-immersion condition, participants used a Meta Quest 2 HMD; in the low-immersion condition, they interacted with the identical simulation on a laptop computer. The virtual patient is controlled by a centralized state machine that manages transitions between predefined exposure and distress states. Transitions between these states trigger synchronized auditory cues (prerecorded patient voice responses) and visual cues (state-specific 3D character animations), which together constitute the visual and auditory feedback that therapists used to guide their decisions. All interaction events (eg, exposure item selections, SUDS checks), exposure transitions, and SUDS values are logged by a persistent session manager and stored in an authentication-protected Firebase cloud database.

##### Treatment Delivery Feedback

**Figure 2.**
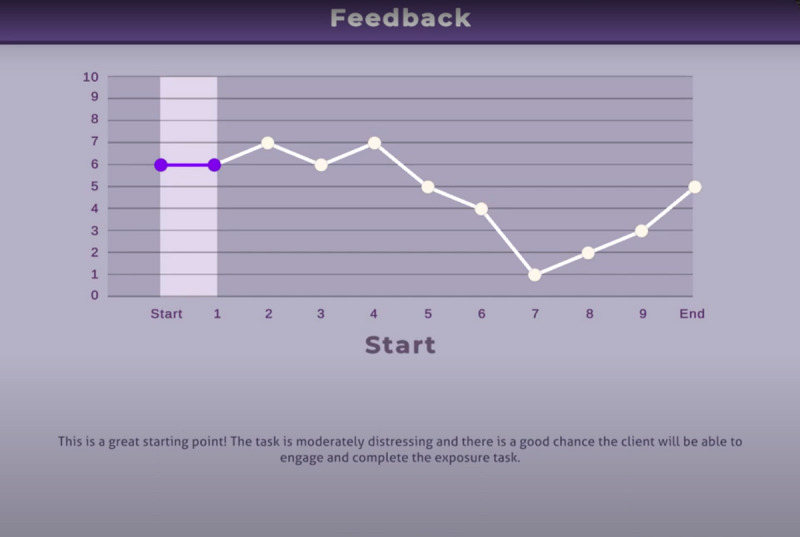
Screen capture of the learning feedback screen provided to therapist learners after completing a SET-VR exposure scenario.

After completing the ninth and final exposure task, the clinician was shown a graph of all their decision points. The feedback screen illustrated the virtual patient’s SUDS levels throughout the experience and at each therapist decision point. As the clinician progressed through the feedback graph, they received specific feedback about each of the decisions they had made (Figure 2).

The program was reset after each use, giving clinicians a blank slate to make new decisions, as if it were the start of a new session (the virtual patient did not “remember” the previous session). The trainer stayed in the room with the participant throughout all 3 repetitions of the SET-VR program to orient the therapist to the headset and controllers and provide technical assistance, if needed. After completing the experiential training, participants received the end-of-study measures, which were completed electronically in the presence of the trainer.

### Ethical Considerations

Study procedures and informed consent documentation were approved by the Brown University Health Institutional Review Board (IRB00004624) in July 2022. All participants provided written consent after meeting with study staff to review the consent documents. As compensation for time spent engaged in study activities, participants received continuing education credits and payment in the form of either cash or an Amazon gift card. Both continuing education credits and monetary compensation (US $300 total) were awarded to participants after they completed all parts of the study. To protect the privacy and confidentiality of participant information, identifying information from the consent process was stored on a secure password-protected server, and all analyses were conducted using a fully deidentified dataset. This is not a secondary analysis, and consent was provided for all reported data. There is no identifying information presented in the analyses or supporting tables and figures. No adverse events or side effects were reported at any point during or after the trial. Study details are reported using CONSORT (Consolidated Standards of Reporting Trials) 2025 (Checklist 1) and CONSORT-EHEALTH (Consolidated Standards of Reporting Trials of Electronic and Mobile Health Applications and Online Telehealth) guidelines [40] (Checklist 2).

### Analytic Plan

Statistical analyses were carried out using SAS version 9.4 1M7. Knowledge, self-efficacy, and TBES were assessed longitudinally and analyzed via binomial generalized estimating equations (GEE). SUS, VP Eval, and TARS were assessed only at the conclusion of the study and analyzed using a binomial generalized linear model. Both sets of analyses used the binomial distribution to constrain the model space to the range of measures being used. The scores were first shifted to have a minimum score of zero by subtracting the minimum score possible. This was used as the binomial number of “successes,” and the range of the score was used as the number of “trials.” Classical sandwich estimation was then used to better incorporate any differences in empirical variances and covariances. Both raw and adjusted (where appropriate) *P* values were presented, as well as unadjusted 95% CIs. For GEEs, 16 different hypotheses were tested using orthogonal linear estimates: (1) changes from baseline conditions in each condition (8 total), (2) high- and low-immersion training conditions compared at each timepoint cross-sectionally (4), and (3) condition comparisons in changes from baseline for each follow-up (4). The Holm test was used to calculate adjusted *P* values, maintaining familywise alpha at .05 across all 16 hypothesis tests in GEEs. For all other models, only 1 hypothesis was tested comparing the conditions. There was no attrition after randomization and participants were required to provide values for all survey items except open-text responses; imputation techniques were not used given the lack of missing data.

## Results

### Baseline Group Comparison (T0)

**Figure 3.**
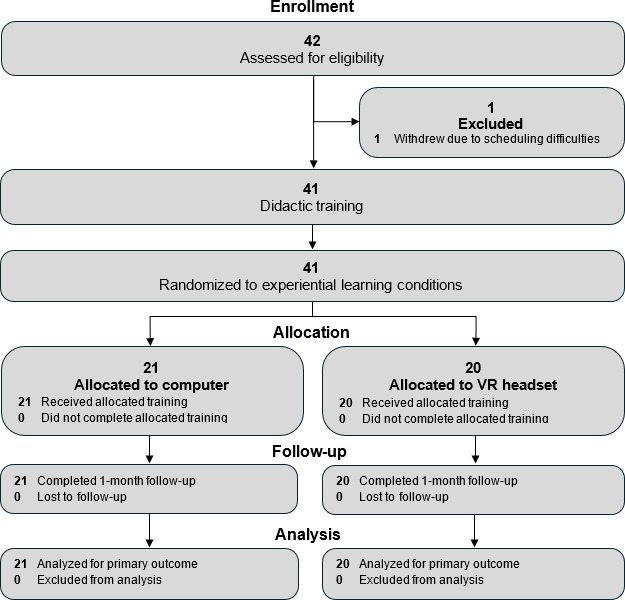
CONSORT (Consolidated Standards of Reporting Trials) diagram of participant flow through a randomized feasibility trial evaluating exposure therapy training for therapists.

The high- and low-immersion training conditions did not exhibit statistically significant differences on measures of exposure knowledge (*t*_117_=1.75, *P*=.08), exposure self-efficacy (*t*_117_=1.90, *P*=.06), and attitudes about exposure therapy (*t*_117_=0.72, *P*=.47) before initiating the workshop training, indicating both groups had similar ratings on key variables prior to initiating the training intervention (Figure 3) [40].

### Post-Didactic Between-Group Differences (T1)

There were no statistically significant differences between the groups on measures of exposure knowledge (*t*_117_=0.93, *P*=0.35), exposure self-efficacy (*t*_117_=1.40, *P*=.17), and attitudes about exposure (*t*_117_=0.00, *P*=.998) following the completion of the didactic portion of training, which suggests both groups demonstrated similar amounts of change in these key variables from baseline to postdidactic timepoints.

### Within-Group Changes From Baseline to Post-Didactic (T0 to T1)

The headset and computer groups exhibited significant improvement in exposure knowledge (*t*_117_=3.16, *P*=.002 and *t*_117_=2.81, *P*=.006, respectively), exposure self-efficacy (*t*_117_=7.37, *P*<.001 and *t*_117_=4.75, *P*<.001, respectively), and attitudes about exposure (*t*_117_=5.68, *P*<.001 and *t*_117_=3.32, *P*=.001, respectively) from baseline to postdidactic timepoints.

### Post-Experiential Between-Group Differences (T2)

There were no significant differences in exposure knowledge (*t*_117_=1.41, *P=*.16), exposure self-efficacy (*t*_117_=1.38, *P*=.17), and attitudes about exposure (*t*_117_=0.11, *P*=.92) at the end of the workshop training.

### Within-Group Changes From Post-Didactic to Post-Experiential (T1 to T2)

**Figure 4.**
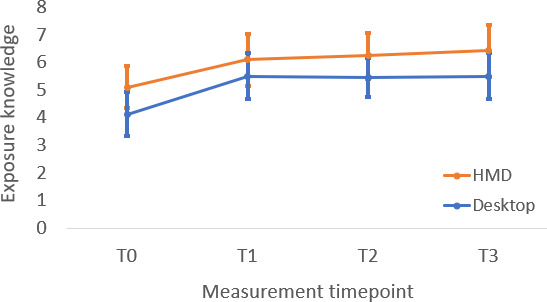
Change in participant exposure knowledge scores across measurement timepoints. HMD: head-mounted display.

**Figure 5.**
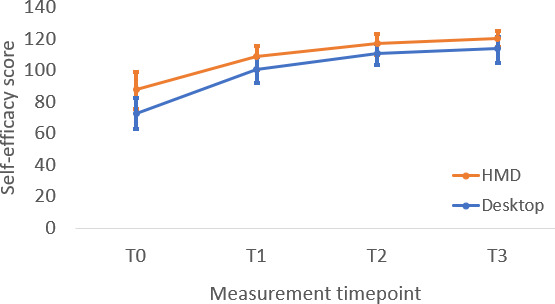
Change in participant exposure self-efficacy scores across measurement timepoints. HMD: head-mounted display.

**Figure 6.**
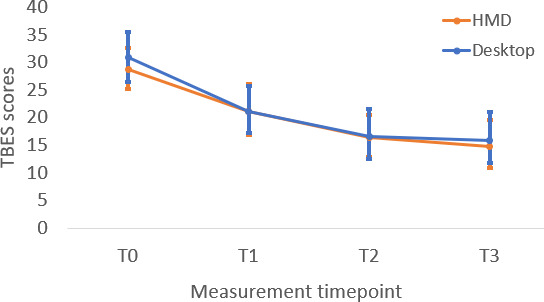
Change in participant beliefs about exposure therapy across measurement timepoints. HMD: head-mounted display; TBES: Therapist Negative Beliefs about Exposure Scale.

The headset and computer groups demonstrated significant improvement in exposure self-efficacy (*t*_117_=5.03, *P*<.001 and *t*_117_=3.58, *P*<.001, respectively) and attitudes about exposure therapy (*t*_117_=3.56, *P*<.001 and *t*_117_=2.5, *P*=.01, respectively) from post-didactic to the end of the workshop timepoints. Neither group demonstrated significant change in exposure knowledge from post-didactic to the end of workshop (*t*_117_=0.19, *P*=.853 and *t*_117_=0.33, *P*=.74, respectively). Taken together, these findings indicate that the experiential portion of training significantly improved therapists’ perceptions of self-efficacy in delivering exposure therapy and their attitudes toward exposure therapy, but it did not significantly improve their knowledge of exposure therapy beyond ratings taken after the completion of didactic activities. Mean knowledge scores at T1 and T2 (Table 2) remained well below the maximum possible score of 12, indicating that the absence of additional knowledge gains from post-didactic to post-experiential was not attributable to a ceiling effect. Instead, this pattern is consistent with the design of the training, in which no new didactic exposure content was introduced during the VR phase (see Figures 4-6).

**Table 2.** Group means and CIs by timepoint and effect sizes for within-group changes across timepoints for primary and secondary outcome measures.

		Within-subject change			
Group and timepoint	Cross-sectional, mean (SD; 95% CI)	*P* value	Adjusted *P* value	Cohen *d*	From
Exposure knowledge					
Headset					
Baseline	34.17 (15.51; 27.37‐40.97)				
Post-didactic	45.83 (16.11; 38.77‐52.89)	.002	.03	0.58	Baseline
Post-experiment	45.42 (13.91; 39.32‐51.52)	.85	.99	−0.04	Post-didactic
Follow-up	45.83 (16.11; 38.77‐52.89)	.99	.99	0.00	Post-didactic
Desktop					
Baseline	42.46 (15.34; 35.90‐49.02)				
Post-didactic	50.79 (18.80; 42.75‐58.83)	.006	.06	0.52	Baseline
Post-experiment	51.98 (16.44; 44.95‐59.01)	.74	.99	0.06	Post-didactic
Follow-up	53.57 (18.92; 45.48‐61.66)	.30	.99	0.19	Post-didactic
Exposure self-efficacy					
Headset					
Baseline	72.35 (23.21; 62.77‐82.53)				
Post-didactic	100.45 (19.22; 91.71‐108.16)	<.001	<.001	1.36	Baseline
Post-experiment	110.70 (15.33; 103.48‐116.67)	<.001	<.001	0.93	Post-didactic
Follow-up	114.00 (19.11; 104.56‐121.04)	<.001	.001	0.74	Post-didactic
Desktop					
Baseline	87.52 (28.45; 75.43‐98.99)				
Post-didactic	108.52 (18.41; 100.04‐115.50)	<.001	<.001	0.88	Baseline
Post-experiment	117.29 (15.44; 109.81‐122.87)	<.001	.004	0.66	Post-didactic
Follow-up	120.38 (11.65; 114.76‐124.63)	<.001	<.001	0.88	Post-didactic
Therapist negative beliefs about exposure scale					
Headset					
Baseline	30.90 (10.49; 26.54‐35.54)				
Post-didactic	21.20 (10.03; 17.18‐25.80)	<.001	<.001	−1.05	Baseline
Post-experiment	16.65 (10.50; 12.58‐21.63)	<.001	.005	−0.66	Post-didactic
Follow-up	15.95 (10.61; 11.87‐21.02)	<.001	.001	−0.75	Post-didactic
Desktop					
Baseline	28.76 (8.96; 25.12‐32.64)				
Post-didactic	21.19 (10.94; 16.92‐26.11)	.001	.01	−0.61	Baseline
Post-experiment	16.33 (9.06; 12.86‐20.48)	.01	.10	−0.46	Post-didactic
Follow-up	14.71 (10.23; 10.89‐19.52)	.002	.02	−0.57	Post-didactic

### Follow-up Between-Group Differences (T3)

There were no significant differences in exposure knowledge (*t*_117_=1.44; *P*=.15), exposure self-efficacy (*t*_117_=1.38; *P*=.17), and attitudes about exposure (*t*_117_=0.39; *P*=.70) after a 1-month follow-up period, indicating comparable maintenance of learning between conditions.

### Within-Group Changes From Post-Experiential to Follow-up (T2 to T3)

The headset and computer groups exhibited durable maintenance of gains during the 1-month follow-up period on measures of exposure knowledge (*t*_117_=0.17; *P*=.87 and *t*_117_=0.49; *P*=.63, respectively), self-efficacy (*t*_117_=1.26; *P*=.21 and *t*_117_=1.50; *P*=.14, respectively), and attitudes about exposure (*t*_117_=0.89; *P*=.38 and *t*_117_=0.94; *P*=.35, respectively).

### Perceptions of the SET-VR Training Simulation (T2)

Ratings of system usability using the SUS measure were statistically significantly higher for the computer group relative to the headset group (*t*_39_=−2.87, *P*=.007); however, usability for both groups (HMD: mean 86.2, SD 9.1, 95% CI 81.68-89.75; desktop: mean 93.4, SD 6.7, 95% CI 89.87-95.80) was rated highly, with both modalities ranking in the upper 96th percentile of usability according to established norms [41]. Therapist perceptions of the virtual patient’s authenticity, degree of engagement, and overall utility as a learning experience, as assessed by the VP Eval measure, were similarly positive in both groups (*t*_39_=0.08; *P*=.93).

### Overall Perceptions of Training (T2)

Ratings of the training processes and their acceptability using the TARS measure were positive. The groups exhibited nearly identical scores on ratings of the training’s acceptability, which assess general acceptability, perceived effectiveness, and appropriateness of the training; however, the computer group reported significantly more positive ratings of training processes (*t*_39_=−2.02; *P*=.05) relative to the headset group, which assessed the extent to which the teaching approach felt helpful.

## Discussion

### Principal Findings

This study tested the feasibility and preliminary effectiveness of a novel exposure therapy training program applying VR technology within an E2E framework. Two delivery modalities with varying degrees of immersion were piloted as different experiential doses of E2E practice to evaluate each method’s usability and impact on key training targets. Clinicians were randomized to training programs of low immersion (desktop computer) and high immersion (HMD) and assessed for changes in exposure knowledge, self-efficacy, and attitudes about exposure therapy.

Our primary outcomes demonstrate that virtual technology is highly usable and effective in engaging relevant therapist-level learning variables during exposure therapy training. Participants rated both virtual programs highly on the SUS, which measures the programs’ ease of use, intuitiveness, and functionality. Likewise, the programs received high scores on the VP evaluation scale, indicating that the virtual patient felt realistic to the therapists. This is especially promising as one challenge in conventional training is the limited ability of role-play to capture the real experience of delivering exposures [12].

Two of the primary training metrics in this study were therapists’ perceptions of their efficacy in delivering exposure therapy and their attitudes about the safety and tolerability of exposure for their clients. Both metrics have been previously identified as therapist-level barriers that limit the frequency and quality of exposure delivery in typical practice [18]. Participants showed significant improvements in the self-efficacy measure, which has been shown to have positive predictive validity for future exposure use [20]. The improvements in TBES scores between T1 (after didactic training) and T2 (after SET-VR) demonstrate that virtual experiential learning further reduced negative beliefs and increased comfort with performing exposures. Notably, these findings were sustained at the 4-week follow-up, indicating potential long-term gains from virtual training. Participants did not show changes in levels of knowledge between T1 and T2. This is to be expected as the SET-VR program focuses on experiential learning with the assumption that the therapist has the necessary theoretical underpinnings. However, our results support virtual experiential learning as a promising option for further development to address key barriers in the current models of exposure therapy training. In contrast, exposure knowledge was included primarily as a control-like outcome to verify that both groups benefited similarly from the didactic workshop and entered the VR phase with comparable background knowledge. This approach is consistent with prior work showing that knowledge about exposure is necessary but far from sufficient to change exposure use or quality, whereas self-efficacy and negative beliefs represent more central barriers to implementation [9].

Interpreted through the E2E lens, these findings suggest that VR-based experiential practice can function as an exposure intervention directed at therapists’ own anxious beliefs about exposure. The additional reductions in negative beliefs and gains in self-efficacy from T1 (post-didactic) to T2 (post-experiential) indicate that repeatedly “doing” exposure with a distressed virtual patient can provide corrective learning beyond what is achievable through didactic instruction alone, even when knowledge is already adequate. This psychotherapy-specific mechanism focus—targeting therapist beliefs that uniquely impede exposure implementation—was central to the rationale for using VR in this trial.

The results from the direct comparison of the 2 delivery conditions addressed our central question about how much immersion is needed for E2E training and did not support our initial hypothesis that the high-immersion condition (HMD) would outperform the low-immersion condition (desktop computer). Participants in both conditions reported comparably high values for the replication of real-life scenarios on VP Eval and satisfaction with the training on the TARS. While both groups gave high ratings on usability (SUS), the low immersion group’s rating was higher at a statistically significant level. As for training outcomes, both conditions improved self-efficacy and negative attitudes without statistically significant between-group differences. Taken together, this finding shows that, at least within this feasibility study, the use of HMD to deliver the virtual training was not superior to the use of a desktop computer in addressing key therapist-level targets, and the lower immersion method may have higher usability. From an E2E perspective, this pattern suggests that, in the context of a brief single-session training, a lower-immersion desktop may already provide sufficient presence and emotional engagement with a distressed virtual patient to facilitate learning about the safety and tolerability of exposure. Higher-immersion HMD may yield incremental benefits only under conditions of greater training “dose” (eg, more repetitions, more complex scenarios) or in subgroups of learners; this is a question that will require a larger, mechanism-focused trial.

In the context of the larger medical education literature, there may also be a task-technology mismatch between virtual exposure therapy delivery and high-immersion VR headset. Prior studies demonstrating a positive correlation between immersion factors and medical education learning outcomes have shown that the effect is most significant in spatial and procedural tasks, such as surgical procedures [42] and physical examinations [43]. Conducting exposure therapy does have procedural elements, like the sequential progression of actions based on branching decision points. However, our participants’ tasks are different from physical procedures in that they do not need a high degree of spatial awareness or physical interaction with the virtual environment, which would be the main advantages of an HMD [44]. Thus, it is possible that visual and auditory stimuli through a desktop computer provided the same necessary input for experiential learning of delivering exposures.

The incremental benefits to learning provided by higher immersion could have been negated by the increased cognitive demands that arise from operating an HMD. Task-irrelevant sensory information or the mental effort required to navigate the less familiar HMD interface could have been distractors from the key experiential task. This would be consistent with the cognitive load theory framework, which states that extraneous cognitive demands reduce mental resources needed for optimal learning [45,46]. Additionally, methodological factors, such as participation selection bias and sample size, may have confounded the detection of between-group differences. One of the most well-studied benefits of high immersion VR is increased user engagement with the material [44]. Given that participants are a self-selected group who are motivated in exposure therapy training, this effect may have been overshadowed. It was anecdotally observed by the study team that participants in the HMD group demonstrated more consistent engagement with the exercise. It is possible that over a longer duration of training, differences in engagement could impact learner outcomes.

Overall, our findings present a basis for further investigation into how immersion functions as an experiential dose parameter within E2E-based psychotherapy training, as well as an opportunity for flexible implementation of VR technology based on factors such as technology literacy, cost-effectiveness, and learner preferences. Most existing studies of VR training in mental health focus on skills, knowledge, and attitudes for specific diagnoses (eg, depression, psychosis) [29]. In contrast, while this study involves a virtual patient with anxiety disorders, its primary aim is to enhance learner outcomes with a specific behavioral health technique (exposure therapy). In this regard, very little research has been done to evaluate procedural learning in VR for behavioral therapy. One notable review identified technology-based methods for training behavioral counseling skills [27]. It found that a mix of virtually created patients and filmed actors was being used. A majority of available outcomes were related to using simulations as opportunities for the repetition of single skills (affirmation, reflective listening, change talk, psychoeducation) [27,47,48], rather than aiming to deliver experiential learning for high-stress clinical scenarios or to address provider beliefs about a treatment modality.

To our knowledge, this is the first study to leverage virtual technology to augment exposure therapy training. The current norm of didactics-focused training is effective in instilling knowledge and, to some extent, improving clinician self-efficacy and negative beliefs [49], as demonstrated even in our single-session training intervention. However, the literature also shows that provider-level barriers continue to limit the optimal dissemination of exposure therapy [13], despite it being one of the most effective tools in psychiatry [18]. Thus, our finding that the supplementation of didactic learning with virtual experiential learning further improves self-efficacy and negative beliefs about exposure therapy suggests a promising area for further investigation to optimize the standardization and scalability of exposure training. This is particularly notable as VR technology in itself offers logistical advantages (accessibility, scalability, reproducibility), the ability to replicate rare or high-risk situations in a safe training space, and near-limitless customizability [50].

Our study design contains notable limitations. There were clear imbalances in the participant demographics. A large majority (90%) of participants were White, non-Hispanic, and female (85%). The gender imbalance may be particularly pertinent, as there have been heterogeneous results regarding the relationship between gender and VR experiences such as immersion, emotional response, and task performance [51]. There is also the potential for selection bias in the recruited participants. While technology-based training interventions are generally well accepted [27], there may be clinicians who prefer other modalities. Those who opted to join this study may be more interested in innovation or technology, which could have influenced their experience or learning outcomes. Additionally, participants were self-selected based on their motivation for exposure therapy training. Although it was not observed in our study, it is possible that cyber sickness and the initial learning curve of new digital platforms may present barriers to engagement in a more general clinician population.

This feasibility trial is designed to assess the effectiveness of SET-VR in addition to didactic training; thus, it is not possible to draw direct comparisons between SET-VR and standard didactic training alone. While a variety of carefully selected metrics were used to assess training efficacy, there are no direct measures of how the quality or frequency of exposures delivered by clinicians changed after SET-VR. Our most reliable proxy is the improvements in exposure self-efficacy, which has previously been shown to have predictive validity in the future likelihood of using exposure therapy [20]. Finally, the current version of SET-VR does not use novel algorithms or adaptive rendering techniques; instead, it uses a controlled, pre-programmed decision structure to standardize the training experience. While this limits technological novelty, it was appropriate for a feasibility trial focused on mechanism-relevant learning outcomes.

This study offers directions for further investigations. In the current trial, the SET-VR architecture intentionally relied on a relatively simple, preprogrammed decision tree and automated SUDS trajectory, rather than more complex adaptive algorithms, to maximize standardization and internal validity for testing immersion and belief change. Future iterations are planned to incorporate more complex interaction logic, including natural language processing–based virtual patient responses and richer affective behaviors. Future iterations will also include a longer training course to see if empirical observations of differences in engagement between immersion levels lead to significant differences in learning. As with other training research, it will be important to evaluate how performance in the exposure simulation sessions correlates with behaviors in actual clinical practice. Noteworthy outcomes could include an increased likelihood of using exposures overall, as well as higher fidelity to the principles of effective exposure therapy [18]. To that end, a larger training trial with practicing clinicians is planned, which will compare decisions during the simulation against coded video tapes of exposure delivery with patients after training to evaluate how performance during training aligns with future delivery.

### Conclusions

The adaptation of VR technology into clinical training offers new opportunities to overcome longstanding limitations in the accessibility, standardization, and experiential quality of exposure training. This study is innovative in applying an explicit “E2E” framework to VR, using simulated exposure sessions to directly target therapists’ anxious beliefs about exposure, and in its examination of immersion as a dose parameter rather than assuming that “more immersive” is inherently better. In contrast to much of the existing VR training literature, which has focused primarily on knowledge and procedural skill acquisition, our findings highlight that both desktop and HMD implementations can produce meaningful changes in self-efficacy and negative beliefs, which are mechanisms that are uniquely tied to exposure underuse in routine care. The lack of clear incremental benefit for HMD over desktop VR suggests that lower-immersion, lower-cost formats may be sufficient to deliver an effective experiential dose of exposure practice, which has important implications for scalability and implementation in community and academic settings. Taken together, these results provide preliminary but encouraging evidence that E2E-guided VR training can enhance conventional didactic training and may be a promising next step in optimizing the standardization and effectiveness of exposure training.

## Supplementary material

10.2196/80087 Checklist 1 CONSORT 2025 checklist.

10.2196/80087 Checklist 2 CONSORT-EHEALTH checklist.

## Abbreviations

CBT: cognitive behavioral therapy
CONSORT: Consolidated Standards of Reporting Trials
CONSORT-EHEALTH: Consolidated Standards of Reporting Trials of Electronic and Mobile Health Applications and Online Telehealth
E2E: exposure to exposure
GEE: generalized estimating equation
HMD: head-mounted display
REDCap: Research Electronic Data Capture
SUDS: Subjective Units of Distress Scale
SUS: System Usability Scale
TARS: Training Acceptability Rating Scale
TBES: Therapist Negative Beliefs about Exposure Scale
VE: virtual environment
VP Eval: Virtual Patient Evaluation
VR: virtual reality
