# Supplementary material for: Enhancing Therapist Training in the Delivery of Exposure Therapy for Individuals with Anxiety Disorders Using Virtual Reality Simulation: Randomized Feasibility Trial
Source: JMIR Med Educ. 2026 May 21;12:e80087. doi: 10.2196/80087 (PMC13193297; doi:10.2196/80087)
Supplement: Checklist 1 [file mededu-v12-e80087-s001.pdf]

Table 1. CONSORT 2025 checklist of information to include when reporting a randomised trial

Section/topic	No	CONSORT 2025 checklist item description	**FROM TRACK CHANGES DOC**
**Title and abstract**			
Title and structured abstract	1a	Identification as a randomised trial	Pg. 1, lines 15-16
	1b	Structured summary of the trial design, methods, results, and conclusions	Pg. 2, lines 2-40
**Open science**			
Trial registration	2	Name of trial registry, identifying number (with URL) and date of registration	Pg. 2, line 41
Protocol and statistical analysis plan	3	Where the trial protocol and statistical analysis plan can be accessed	[abridged protocol is available on ClinicalTrials.org]
Data sharing	4	Where and how the individual de-identified participant data (including data dictionary), statistical code and any other materials can be accessed	Pg. 29, lines 27-28
Funding and conflicts of interest	5a	Sources of funding and other support (eg, supply of drugs), and role of funders in the design, conduct, analysis and reporting of the trial	Pg. 29, line 24
	5b	Financial and other conflicts of interest of the manuscript authors	Pg. 29, lines 20-21
**Introduction**			
Background and rationale	6	Scientific background and rationale	Pg. 3 (line 1) to Pg. 7 (line 12)
Objectives	7	Specific objectives related to benefits and harms	Pg. 6, lines 18-24
**Methods**			
Patient and public involvement	8	Details of patient or public involvement in the design, conduct and reporting of the trial	
Trial design	9	Description of trial design including type of trial (eg, parallel group, crossover), allocation ratio, and framework (eg, superiority, equivalence, non-inferiority, exploratory)	Pg. 6, lines 15-18; pg. 9, line 17
Changes to trial protocol	10	Important changes to the trial after it commenced including any outcomes or analyses that were not prespecified, with reason	N/A

Section/topic	No	CONSORT 2025 checklist item description	FROM TRACK CHANGES DOC
Trial setting	11	Settings (eg, community, hospital) and locations (eg, countries, sites) where the trial was conducted	Pg. 7, lines 6-7; pg. 9, lines 12-14
Eligibility criteria	12a	Eligibility criteria for participants	Pg. 7, lines 9-12
	12b	If applicable, eligibility criteria for sites and for individuals delivering the interventions (eg, surgeons, physiotherapists)	N/A
Intervention and comparator	13	Intervention and comparator with sufficient details to allow replication. If relevant, where additional materials describing the intervention and comparator (eg, intervention manual) can be accessed	Pgs. 10-13 (“Procedures” section)
Outcomes	14	Prespecified primary and secondary outcomes, including the specific measurement variable (eg, systolic blood pressure), analysis metric (eg, change from baseline, final value, time to event), method of aggregation (eg, median, proportion), and time point for each outcome	Pgs. 7-9 (“Measures” section), Table 1
Harms	15	How harms were defined and assessed (eg, systematically, non-systematically)	Pg. 13, lines 19-20
Sample size	16a	How sample size was determined, including all assumptions supporting the sample size calculation	Pg. 7, lines 1-2
	16b	Explanation of any interim analyses and stopping guidelines	N/A
Randomisation:			
Sequence generation	17a	Who generated the random allocation sequence and the method used	
	17b	Type of randomisation and details of any restriction (eg, stratification, blocking and block size)	Parallel allocation, no blocking
Allocation concealment mechanism	18	Mechanism used to implement the random allocation sequence (eg, central computer/telephone; sequentially numbered, opaque, sealed containers), describing any steps to conceal the sequence until interventions were assigned	Pg. 9, lines 17-18; pg. 10, lines 1-2
Implementation	19	Whether the personnel who enrolled and those who assigned participants to the interventions had access to the random allocation sequence	Pg. 10, lines 1-2 and 17-19
Blinding	20a	Who was blinded after assignment to interventions (eg, participants, care providers, outcome assessors, data analysts)	Pg. 10, lines 17-20

Section/topic	No	CONSORT 2025 checklist item description	FROM TRACK CHANGES DOC
	20b	If blinded, how blinding was achieved and description of the similarity of interventions	N/A
Statistical methods	21a	Statistical methods used to compare groups for primary and secondary outcomes, including harms	Pg. 13 (line 22) to pg. 14 (line 11)
	21b	Definition of who is included in each analysis (eg, all randomised participants), and in which group	Pg. 14, lines 6-9
	21c	How missing data were handled in the analysis	Pg. 14, lines 11-13
	21d	Methods for any additional analyses (eg, subgroup and sensitivity analyses), distinguishing prespecified from post hoc	N/A
**Results**			
Participant flow, including flow diagram	22a	For each group, the numbers of participants who were randomly assigned, received intended intervention, and were analysed for the primary outcome	Pg. 9, lines 14-17; Figure 1 (CONSORT)
	22b	For each group, losses and exclusions after randomisation, together with reasons	Figure 1 (CONSORT)
Recruitment	23a	Dates defining the periods of recruitment and follow-up for outcomes of benefits and harms	Pg. 7, lines 5-6
	23b	If relevant, why the trial ended or was stopped	N/A
Intervention and comparator delivery	24a	Intervention and comparator as they were actually administered (eg, where appropriate, who delivered the intervention/comparator, how participants adhered, whether they were delivered as intended (fidelity))	Pg. 10, lines 17-23; pg. 11, lines 2-4; pg. 13, lines 1-7
	24b	Concomitant care received during the trial for each group	N/A
Baseline data	25	A table showing baseline demographic and clinical characteristics for each group	[available on ClinicalTrials.gov]
Numbers analysed, outcomes and estimation	26	For each primary and secondary outcome, by group:<br>● the number of participants included in the analysis<br>● the number of participants with available data at the outcome time point<br>● result for each group, and the estimated effect size and its precision (such as 95% confidence interval)<br>● for binary outcomes, presentation of both absolute and relative effect size	Figure 1 (CONSORT), pgs. 14-17 (Results section), Table 2

Section/topic	No	CONSORT 2025 checklist item description	**FROM TRACK CHANGES DOC**
Harms	27	All harms or unintended events in each group	Pg. 13, lines 19-20
Ancillary analyses	28	Any other analyses performed, including subgroup and sensitivity analyses, distinguishing pre-specified from post hoc	N/A
**Discussion**			
Interpretation	29	Interpretation consistent with results, balancing benefits and harms, and considering other relevant evidence	Pg. 18 (line 9) to pg. 22 (line 6)
Limitations	30	Trial limitations, addressing sources of potential bias, imprecision, generalisability, and, if relevant, multiplicity of analyses	Pg. 22, (line 7) to pg. 23 (line 3)
